# Growth differentiation factor-11 supplementation improves survival and promotes recovery after ischemic stroke in aged mice

**DOI:** 10.18632/aging.103122

**Published:** 2020-05-04

**Authors:** Jacob Hudobenko, Bhanu Priya Ganesh, Jianjun Jiang, Eric C. Mohan, Songmi Lee, Sunil Sheth, Diego Morales, Liang Zhu, Julia K. Kofler, Robia G. Pautler, Louise D. McCullough, Anjali Chauhan

**Affiliations:** 1 Department of Neurology, McGovern Medical School, University of Texas Health Science Center at Houston, Houston, TX 77030, USA; 2 Baylor College of Medicine, Houston, TX 77030, USA; 3 University of Connecticut Health Science Center, Farmington, CT 06030, USA; 4 Biostatistics and Epidemiology Research Design Core, Center for Clinical and Translational Sciences, University of Texas Health Science Center at Houston, Houston, TX 77030, USA; 5 Department of Pathology, University of Pittsburgh, Pittsburgh, PA 15213, USA; 6 Memorial Hermann Hospital, Texas Medical Center, Houston, TX 77030, USA

**Keywords:** aging, GDF11, stroke, gliosis, White matter integrity

## Abstract

Growth differentiation factor (GDF) 11 levels decline with aging. The age-related loss of GDF 11 has been implicated in the pathogenesis of a variety of age-related diseases. GDF11 supplementation reversed cardiac hypertrophy, bone loss, and pulmonary dysfunction in old mice, suggesting that GDF11 has a rejuvenating effect. Less is known about the potential of GDF11 to improve recovery after an acute injury, such as stroke, in aged mice. GDF11/8 levels were assessed in young and aged male mice and in postmortem human brain samples. Aged mice were subjected to a transient middle cerebral artery occlusion (MCAo). Five days after MCAo, mice received and bromodeoxyuridine / 5-Bromo-2'-deoxyuridine (BrdU) and either recombinant GDF11 or vehicle for five days and were assessed for recovery for one month following stroke. MRI was used to determine cerebrospinal fluid (CSF) volume, corpus callosum (CC) area, and brain atrophy at 30 days post-stroke. Immunohistochemistry was used to assess gliosis, neurogenesis, angiogenesis and synaptic density. Lower GDF11/8 levels were found with age in both mice and humans (p<0.05). GDF11 supplementation reduced mortality and improved sensorimotor deficits after stroke. Treatment also reduced brain atrophy and gliosis, increased angiogenesis, improved white matter integrity, and reduced inflammation after stroke. GDF11 may have a role in brain repair after ischemic injury.

## INTRODUCTION

With aging, many changes in structural and biological processes occur, and in part can be explained by alterations in circulating systemic factors. Parabiosis studies, using young and aged mice have identified several systemic factors that are highly expressed in young mice and can reverse features of aging [1]. Growth differentiation factor 11 (GDF11) is a circulating member of the transforming growth factor-beta (TGF-β) superfamily that has been shown to improve cardiac function and neurogenesis in old mice [2, 3]. GDF11 is highly homologous to GDF8, myostatin, which negatively regulates skeletal muscle mass [4]. GDF11 has a controversial history as studies have reported both a protective and a detrimental role of GDF11 in age-related diseases [5–10]. Earlier studies have demonstrated that serum, spleen and kidney levels of GDF11/8 decline with age [11] and restoration of GDF11 reverses age-associated cardiac hypertrophy, skeletal muscle dysfunction, hippocampal vascularity and increases neural stem-cell proliferation [2, 3, 7, 12]. Additionally, exogenous GDF11 treatment led to improvements after kidney injury in older animals [9] and resulted in anti-senescent effects and reduced alveolar damage in an animal model of emphysema [13]. GDF11 treatment reduced inflammation, oxidative stress, and apoptosis in an experimental intracerebral hemorrhage model in old rats [14]. Administration of GDF11 reduced cognitive deficits and amyloid deposition in a mouse model of Alzheimer’s disease [15] and resulted in vascular remodeling and neurogenesis in older wild-type mice [12]. In ischemic stroke, GDF11 treatment improved functional outcome and stimulated neurogenesis and angiogenesis in rats and mice [16, 17] by upregulating the levels of brain-derived neurotrophic factor (BDNF), angiopoietin-2 and vascular endothelial factor receptor-2 however; these studies were only conducted in young animals. Recent studies found a decline in circulating levels of GDF11 in aged individuals [18, 19]. Furthermore, in large clinical cohorts, higher levels of GDF11 or GDF11/8 were closely associated with a lower risk of cardiovascular events and mortality [20, 21]; suggesting GDFD11 had a cardioprotective role in humans. However, other studies have found that GDF11 may inhibit skeletal muscle regeneration and had no effect on cardiac hypertrophy [6, 8], leading to considerable controversy regarding the utility of GDF11 supplementation as a therapeutic strategy. However, many of these studies used different routes of administration and dosing [2, 6, 10].

Stroke is a leading cause of mortality and disability in the United States and the majority of strokes occur in older adults [22, 23]. Age-related changes in regenerative processes could be partially responsible for the poorer recovery seen in the elderly [24]. Declining levels of BDNF [25], decreased angiogenesis [26] and neurogenesis [27], enhanced or altered inflammatory responses [28], and increased astrocyte reactivity and gliosis [29] contribute to poorer recovery after injury in the aged brain. As GDF11 signaling decreases with age and stroke is primarily a disease of the elderly, examining its effects in aged models of stroke increases translational relevance. Therefore, in this study, we investigated the expression of GDF11 in aging, in mice and humans. The effects of exogenous GDF11 given during stroke recovery in aged animals were also examined.

## RESULTS

### Brain GDF11 declines with age in mice and humans

**Figure 1:**
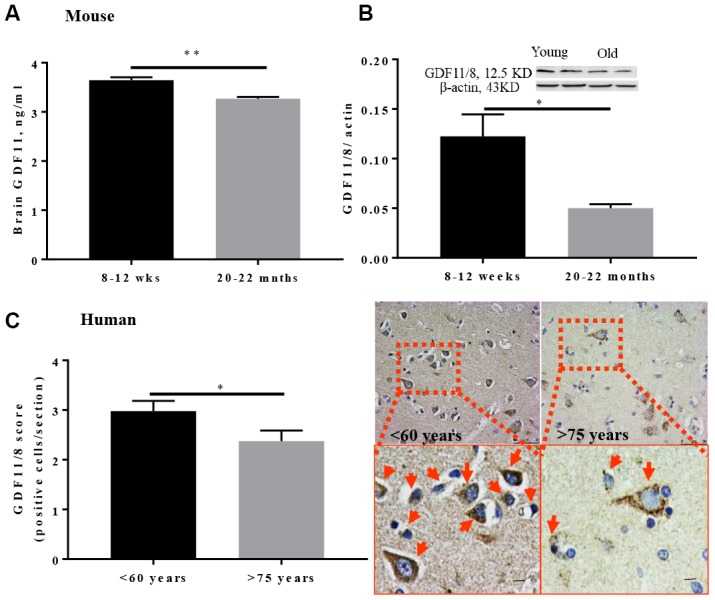
**GDF11/8 levels decline with age in both mice and humans.** (**A**) ELISA shows GDF11 levels decline with age in the brain of aged mice (20-22-month-old), (**B**) reduced brain GDF11/8 expression in old mice was confirmed by Western blotting, (**C**) brain GDF11/8 (brown) positive cells decline with age in older humans. Hematoxylin (blue) counterstain. Mouse studies, n=5, human. Magnification 20X, n=8-9, *p<0.05, **p<0.01.

To determine whole-brain GDF11 levels, young (8-12 weeks) and aged (20-22 months) male mice were euthanized and brains were collected for western blot and ELISA. Brain GDF11 levels were significantly lower in older mice (p<0.01, Figure 1A) as assessed by ELISA. Brain GDF11/8 (12.5 kDa) expression was also assessed by Western blot and was significantly lower in old (p<0.05, Figure 1B) versus young mice. GDF11/8 positive cells were significantly decreased (p<0.05, Figure 1C) in individuals above 75 years as compared to those <60 years of age.

### Recombinant GDF11 treatment improved early neurological recovery and reduced brain inflammation after transient ischemic stroke

**Figure 2:**
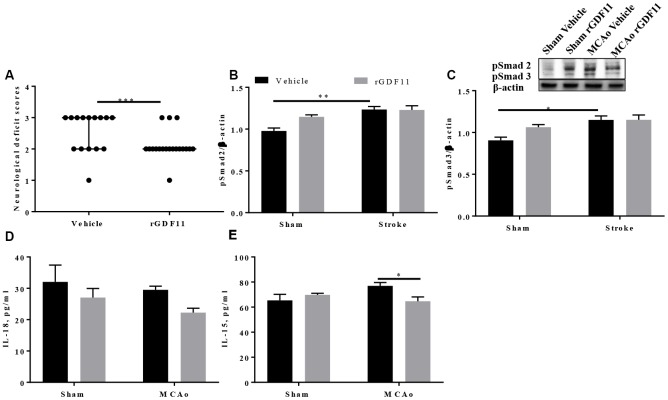
**Recombinant GDF11 treatment improved early neurological recovery by reducing brain inflammation after transient ischemic stroke.** (**A**) a decrease in NDS at day 10 post MCAo, (**B**) pSmad2 and (**C**) psmad3 expression in the vehicle and rGDF11 treated mice at day 10 post-stroke, (**D**) decrease in brain IL-18 and (**E**) IL-15 levels in the MCAorGDF11 treated mice at day 10 post-stroke n=3-7,*p<0.05.

It is unknown if the age-related loss of GDF11 contributes to the poor post-stroke repair seen in aged animals. To determine if GDF11 altered early neurological outcomes in aged male animals after stroke, we administered recombinant growth differentiation factor-11 (rGDF11) five days after middle cerebral artery occlusion (MCAo) for 5 days. At day 10 post-stroke, a significant decrease (p<0.05, Figure 2A) in the neurological deficit score was seen in the GDF11 treated mice suggesting an earlier recovery of neurological deficits. GDF11 acts by phosphorylation and activation of Smad2 and Smad3 [11, 30]. A slight increase in the brain pSmad2 and pSmad3 expression was seen in the sham mice treated with rGDF11 compared to sham vehicle, this did not reach statistical difference. An increase in brain pSmad2 and pSmad3 expression was seen between sham and MCAo vehicle-treated groups (Figure 2B, 2C). No difference between sham rGDF11 and MCAo rGDF11 treated mice was seen in brain pSmad2 and pSmad3 expression. A decrease in brain IL-18 levels with exogenous GDF11 was seen (p<0.05, Figure 2D). Furthermore, an interaction between surgery and treatment was seen in brain IL-15 levels (p<0.05). A significant decrease (p<0.05) in brain IL-15 levels was seen in the MCAo rGDF11 treated mice at day 10 post-stroke (Figure 2E) suggesting that rGDF11 reduced brain inflammation.

### Exogenous GDF11 administration led to decreased mortality, reduced brain tissue loss, and increased motor activity at 30 days after experimental stroke

**Figure 3:**
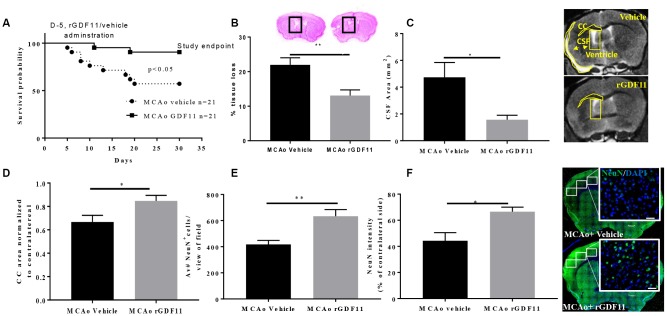
**Reduced tissue loss and mortality after exogenous GDF11 treatment in old MCAo mice.** (**A**) survival probabilities, GDF11 treatment led to (**B**) less brain tissue loss, (**C**) decrease in cerebrospinal fluid (CSF) area, and preserved (**D**) corpus callosum (CC) area. An increase in (**E**) NeuN count and (**F**) intensity with rGDF11 treatment was seen at 30 days post-stroke. Magnification 20X. Scale bar 20 μm. n=5-8,*p<0.05, **p<0.01.

There was a decline in the body weights in all stroke cohorts at day 7 post-stroke compared to respective sham-treated mice (p<0.05) which gradually recovered by day 30 (Supplementary Figure 1). We observed a significant increase in the survival probability between the MCAo rGDF11 group as compared to the MCAo vehicle group (p<0.05, Figure 3A). The mortality in the MCAo cohort treated with vehicle was as follows; day 5 (1 mouse), day 6 (1 mouse), day 8 (2 mice), day 10 (1 mouse), day 13 (1 mouse), day 18 (1 mouse) day 19 (1 mouse) and day 20 (1 mouse) after stroke. In the MCAo cohort treated with rGDF11, mortality was observed on day 11 and 19. In total nine mice in the vehicle group and two mice in GDF11 died respectively. There was no morality in sham mice.

Cresyl violet staining showed a significant reduction in brain tissue loss in the rGDF11 group at 30 days post-stroke compared to vehicle mice (p<0.01, Figure 3B). This was confirmed by MRI. MRI showed a decrease in the CSF area (p<0.05, Figure 3C) and an increase in the CC area (p<0.05, Figure 3D) in the MCAo rGDF11 group as compared to the MCAo vehicle-treated mice. Thirty days post-MCAo, the number of NeuN^+^ cells and intensity was higher in the MCAo rGDF11 group as compared to MCAo vehicle (p<0.05, Figure 3E, 3F) suggesting a protective role of GDF11 during stroke recovery.

**Figure 4:**
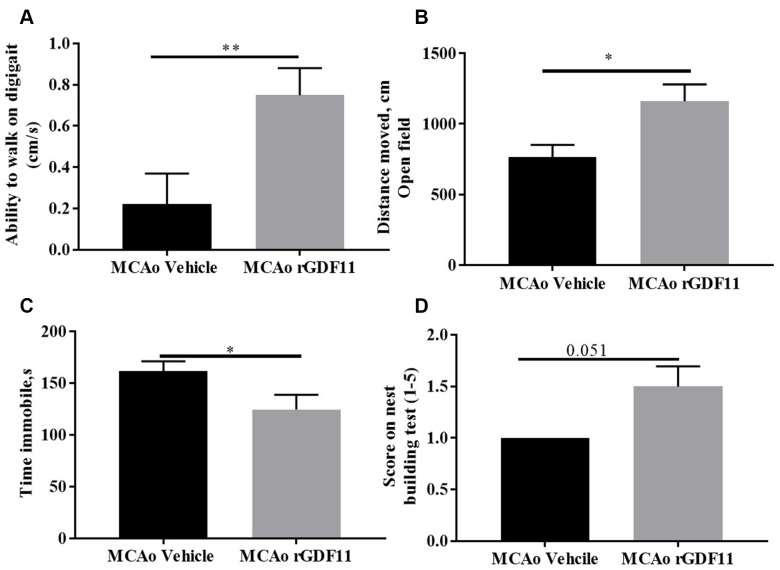
**Improvement in motor function on day 30 with rGDF11 treatment.** (**A**) improved gait function as assessed by the digigait at day 14, (**B**) increase in distance moved in the open field at day 30 post-MCAo, (**C**) reduction in time immobile, (**D**) nest-building score at day 30 post MCAo in aged mice. n=6-12 *p<0.05, ***p<0.001.

At day 14 post-stroke, an increase (p<0.05, Figure 4A) in the ability to walk, evaluated by digigait testing, was seen in rGDF11-treated mice as compared to vehicle-treated animals. An increase (p<0.05, Figure 4B) in the total distance moved in open field task was also seen in the rGDF11 treated cohort at day 30 post-stroke, demonstrating an improvement in overall locomotor activity. Mice in the rGDF11 group were more mobile during the tail suspension task compared to the stroke vehicle group suggesting a role of GDF11 in depressive behavior (Figure 4C). Nest building ability, a measure of sensorimotor and cognitive function [31], was not significantly different between the groups (p=0.05); although the nest building score was higher in the rGDF11 treated mice as compared to vehicle-treated mice (Figure 4D). No difference between sham vehicle and sham rGDF11 mice was seen in nest building, the ability to walk on digigait and distance moved on an open field (Supplementary Figure 2). There was a significant decrease in the time immobile on tail suspension task seen in the sham rGDF11 treated mice as compared to sham vehicle (Supplementary Figure 2D) suggesting GDF11 might reduce depressive phenotypes.

### Exogenous GDF11 reduces gliosis and increases angiogenesis after stroke

**Figure 5:**
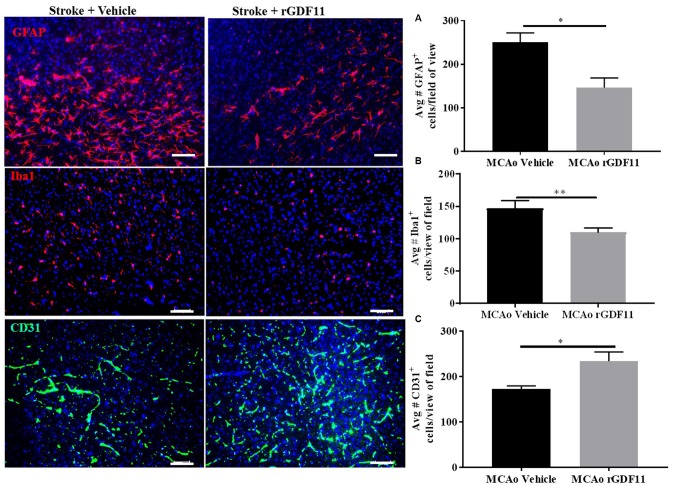
**Exogenous GDF11 reduced gliosis and increased CD31^+^ endothelial cells.** (**A**) decline in GFAP ^+^ and (**B**) Iba-1 ^+^ cells in MCAo GDF11 group at day 30 post-stroke, (**C**) increase in CD31^+^ endothelial cells in the MCAo GDF11 cohort at day 30 after stroke. Magnification 20X. Scale bar 20μm. n=5. *p<0.05.

**Figure 6:**
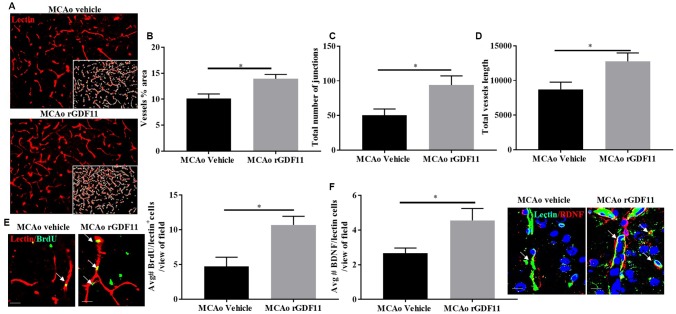
**Exogenous GDF11 increased angiogenesis in older mice after stroke.** (**A**) representative images showing lectin stain and inset shows quantitatively measured region using Angio tool software (Magnification 20X), (**B**) vessel percentage area, (**C**) total number of junctions, (**D**) total vessel length was increased in the MCAo rGDF11 treated older mice, (**E**) representative images showing BrdU and lectin stain in the treatment groups, BrdU/lectin^+^ cells and BDNF/lectin^+^ cells in the treatment groups, (**F**) increase in BDNF/lectin^+^ cells in the rGDF11MCAo group. Magnification 20X (Lectin/BrdU^+^ cells) magnification 63X (Lectin/BDNF^+^ cells). Scale bar 20μm. n= 5-6, *p<0.05.

The immunoreactivity of glial fibrillary acidic protein (GFAP), ionized calcium-binding adaptor protein (Iba-1), and CD31 was assessed 30 days after stroke to investigate if rGDF11 had effects on gliosis, or angiogenesis respectively. No difference between the average number of GFAP, Iba-1, and CD31^+^ endothelial cells was seen between sham vehicle and sham rGDF11 mice (Supplementary Figure 3). There was a significant decrease (p<0.05, Figure 5A) in GFAP^+^ cells in the peri-infarct area in the rGDF11 treated mice compared to vehicle-treated stroke mice, reflecting reduced astrogliosis after exogenous rGDF11 treatment. A reduced (p<0.05, Figure 5B) number of Iba-1^+^ cells was seen with exogenous rGDF11 treatment, suggesting a decrease in microglial activation in treated mice. Treatment with rGDF11 increased CD31^+^ endothelial cells (p<0.05, Figure 5C) as compared to vehicle-treated mice post-stroke. We observed an increase in the vessel percentage area (p<0.05, Figure 6B), the total number of vessel branch points (p<0.05, Figure 6C) and total vessel length (p<0.05, Figure 6D) in the rGDF11-treated mice as compared to the vehicle-treated group. Furthermore, an increase (p<0.05, Figure 6E) in BrdU/lectin^+^ cells was observed in the GDF11 group as compared to the vehicle group at day 30 post-stroke, reflecting an increase in angiogenesis. An increase (p<0.05, Figure 5F) in the number of BDNF/lectin^+^ cells was seen in the MCAo rGDF11 mice as compared to the MCAo vehicle group. There was an increase in BrdU^+^ cells after rGDF11 treatment (p<0.05) and stroke (p<0.05) in the subventricular zone (SVZ) (Supplementary Figure 4). An increase in the BrdU^+^ cells after and stroke (p<0.05) was seen in the hippocampus. There was no difference in DCX^+^ BrdU^+^ cells in the SVZ or hippocampus (Supplementary Figure 4) between rGDF11 and vehicle-treated groups at 30 days post-stroke.

### GDF11 treatment reduces white matter injury and restores presynaptic plasticity

**Figure 7:**
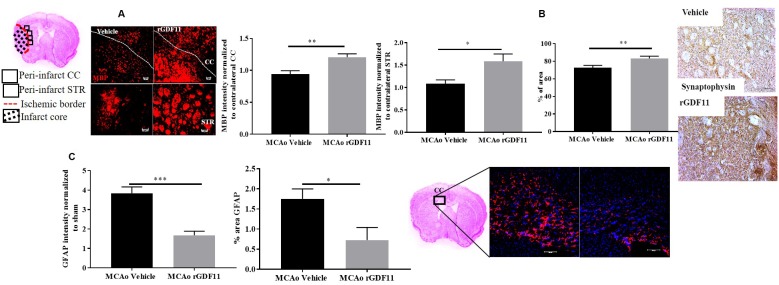
**GDF11 treatment restores white matter integrity and synaptic plasticity at day 30 post-MCAo.** (**A**) increase in MBP intensity in the CC and peri-infarct striatum, (**B**) increase in synaptophysin immunostaining, (**C**) decrease in GFAP^+^ cells in CC at day 30 post MCAo in aged mice. Magnification 20X. Scale bar 50μm. n=5-7. *p<0.05, **p<0.01.

We investigated the role of GDF11 on synaptic plasticity, axonal damage, and demyelination in the corpus callosum and peri-infarct regions. Synaptophysin is a reliable marker for axonal damage, presynaptic plasticity, and synaptogenesis [32] and MBP is a marker of white matter damage [33]. Recombinant GDF11 treatment increased MBP intensity in the CC and peri-infarct striatum (STR) (p<0.05, Figure 7A). The expression of synaptophysin was increased in the rGDF11 treated group compared to vehicle-treated stroke mice (p<0.05, Figure 7B). Additionally, a decrease (p<0.05, Figure 7C) in the GFAP intensity and percentage area GFAP in the CC was observed in the rGDF11 treated group compared to vehicle-treated stroke mice.

## DISCUSSION

GDF11 and GDF8 have 90% protein sequence homology [4]. Egerman et al. and Smith et al. reported that the reagents used to identify GDF11 also recognize GDF8 [5, 8]. By using the GDF11 specific assay, Egerman et al. showed a trend towards an increase in the GDF11 levels with aging in both rats and humans [5]. However, others have reported that an increase in the GDF11 reported by Egerman et al., is not GDF11 but immunoglobulin light chain (band ~25 kDa by Western blot) [11]. We found that brain GDF11/8 levels decreased with age in mice, which is consistent with others that showed a tissue-specific GDF11 decline with age [3, 7, 9]. We used the only available antibody for GDF11/8, which detects both GDF11 and GDF8. We confirmed the age-related loss in GDF11 with a GDF11 ELISA kit. This also showed a reduction in brain GDF11 levels with aging. A previous study by Lu et al., effectively demonstrated the neuroprotective potential of GDF11 in stroke [16]. This study was performed in young mice that underwent a distal middle cerebral artery model that leads to much smaller infarcts than that seen after proximal middle cerebral artery occlusion. We used the proximal MCAo model, as this is the most common stroke seen in older clinical populations [34]. We administered GDF11 at day 5 post-ischemia, by this time; the infarct is fully mature, as we wanted to evaluate the efficacy of GDF11 on brain recovery rather than its potential neuroprotective effects. We observed a reduction in brain ventricle size; CSF area and reduced mortality in the GDF11 treated cohort thirty days post-MCAo.

GDF11 functions by phosphorylation and activation of Smad2 and Smad3 similar to TGF-β [11, 30]. Activation of TGF-β signaling has diverse functions including an increase in angiogenesis and neurogenesis [35–38]. However, the cellular responses to activation of Smad2/3 are context-dependent. The presence or absence of transduction regulatory factors, transcription factors and epigenetic status of the cell alters the ligand binding [39]. We observed a slight increase in the brain expression of pSmad2 and pSmad3 after rGDF11 treatment in sham mice, but this was not significant.

The initial ischemic injury is followed by secondary inflammatory responses and secretion of pro-inflammatory cytokines that promote cerebral injury [40–42]. IL-18 is secreted by microglia and monocytes/macrophages in the infarcted cortex after ischemic stroke rodents and humans [41, 43]. A decrease in the brain IL-18 levels with the rGDF11 treatment was observed in this study. Furthermore, we observed a decrease in brain IL-15 levels in the MCAo rGDF11 treated mice. IL-15 expression is increased in astrocytes and microglia after injury and worsens functional outcomes after stroke [42, 44]. Hence, these findings suggest that GDF11 may have an anti-inflammatory role after stroke.

As reported previously, stroke induces activation and proliferation of glial cells resulting in neuroinflammation [45, 46]. In our study, rGDF11 treatment attenuated the proliferation of glial cells in old mice after stroke. A decrease in brain IL-15 and IL-18 levels at day 10 along with reduced gliosis at day 30-post stroke could partially explain the protective role of GDF11. A recent study highlighted that GDF11 treatment reduced both mRNA and protein levels of GFAP and Iba1 in a mouse model of Alzheimer’s disease, suggesting a role of GDF11 in attenuation of neuroinflammation [15]. Previous studies have confirmed that GDF11 also stimulates vascular remodeling and angiogenesis [12, 47] in older animals. Moreover, a recent study has highlighted the pro-angiogenic effect of rGDF11 in early Alzheimer’s disease model [15]. We observed an increase in CD31 with GDF11 treatment in older stroke animals. Additionally, an increase in BrdU/lectin^+^ cells was observed in the MCAo rGDF11 group reflecting angiogenesis. This is consistent with other studies in ischemia/reperfusion models where GDF11 increased endothelial progenitor cells and increased angiogenesis in ischemic hearts [47] as well as after cerebral injury [17]. We observed an increase in BDNF/lectin^+^ cells in mice treated with rGDF11 suggesting GDF11 could be partially responsible for the angiogenic effects [2, 16]. An earlier study showed that rGDF11 does not cross the blood-brain barrier but instead, induced secretion of CNS active factors *in vitro* and stimulated endothelial cells and increased neurogenesis [2]. Taken together, the CNS protective effects observed in our study could partly be explained by the reduction in neuroinflammation (during both the early and chronic phase of injury) and endothelial cells could be potential cellular targets for GDF11, an area we are actively pursuing.

White matter is vulnerable to ischemia-reperfusion injury and hence damage to white matter is associated with long-term neurological deficits [48]. Inflammation and oxidative stress-induced after ischemic stroke contribute to axonal demyelination, white matter damage and neurobehavioral deficits [49, 50]. We observed a restoration of MBP and synaptophysin levels in the MCAo GDF11 group in the peri-infarct area and CC at 30 days after stroke. Additionally, a decrease in the GFAP intensity and percentage area in the CC was observed at 30 days in the MCAo rGDF11 treated mice. Previous studies have shown that chronic astrogliosis within the white matter was accompanied by pro-inflammatory signaling and resulted in white matter damage and cognitive impairment in mice [48, 51]. This protective effect on white matter integrity and the observed improvement behavioral changes could be partially explained by the reduction of astrogliosis by rGDF11. However further studies are needed to validate the role of GDF11 on the repair and recovery mechanisms in stroke injury.

Our results demonstrated that GDF11 treatment did not affect neurogenesis, although others have reported an increase in neurogenesis with GDF11 in older uninjured mice as well as after cerebral ischemic injury in young animals. This is likely due to the more prolonged treatment (30 days in uninjured older mice) and that young mice have higher neurogenic potential compared to aged mice after stroke [27]. This is the first study that examines GDF11 replacement in the aged brain after stroke, and the drive for, or the timing of, post-stroke neurogenesis may be altered in the aged brain. Although we did observe an increase in BrdU^+^ cells in the rGDF11 treated mice, it is possible that the shorter duration or dose of GDF11 used in our study was not sufficient to stimulate the formation of new neurons in stroke animals or that the administration of BrdU was timed incorrectly.

Our study has several limitations. First, we did not observe neurogenesis with GDF11 supplementation as reported by others [12, 16]. Secondly, although we show rGDF11 administration in the recovery phase is beneficial, additional studies testing how GDF11 modulates gliosis and blood-brain barrier recovery after stroke are needed, as are studies examining both sexes. We show that GDF11 treatment is protective in the older stroke mice and significantly reduced mortality, but the underlying mechanisms could be manifold. Lack of GDF11 specific inhibitors and GDF11 knockout animals limit our understanding of the neuroprotective mechanism of GDF11 in aging and stroke.

In summary, brain GDF11/8 levels decline with age in both mice and humans. Five days of GDF11 administration to old male mice initiated five days after stroke reduced brain tissue loss, mortality and improved sensorimotor outcomes at 30 days. Exogenous GDF11 decreased gliosis and stimulated angiogenesis. Furthermore, GDF11 treatment improved white matter integrity post-stroke.

## MATERIALS AND METHODS

### Study subjects

C57BL/6J young (8-12 weeks) and old (20-22 months) male mice were pair-housed in a specific pathogen-free facility (light cycle 12/12 h light/dark). Food and water were provided *ad libitum*. All animal procedures were performed in accordance with NIH guidelines for the care and use of laboratory animals and approved by the Animal Care Committee of the University of Texas Health Science Center at Houston, McGovern Medical School.

All human tissue samples were obtained from the University of Pittsburgh neurodegenerative brain bank with appropriate ethics committee approval (Committee for Oversight of Research and Clinical Training Involving Decedents). provides the demographic details for the human controls.

### Experimental groups

To investigate baseline changes in GDF11 levels with age, young and old mice were euthanized and serum and brain GDF11 levels were assessed. For the long-term recovery study (30 days) mice underwent MCAo surgery and were randomly divided into four groups, sham vehicle, sham rGDF11, MCAo vehicle, and MCAo rGDF11**.** To investigate the role of rGDF11 in early stroke recovery, mice were randomly divided in the sham vehicle, sham rGDF11, MCAo vehicle, and MCAo rGDF11 and euthanized at 10-day post MCAo

### Middle cerebral artery occlusion (MCAo) and treatment

Aged mice underwent transient focal ischemia under isoflurane anesthesia for 1 hour by occlusion of the right middle cerebral artery (MCA) [52] details of MCAo procedure are provided in Supplementary methods.

The body temperature was measured with a rectal probe and maintained at ≥ 36.5 ^o^C using a feedback-controlled heating pad. Cerebral blood flow (CBF) was measured by laser Doppler flowmeter; animals that had ≥ 80% drop in CBF were included in the study. All mice were given subcutaneous injections of 0.9% sodium chloride twice a day for 7 days and were provided with wet mash in their cages. Body weight was recorded daily for the duration of the experiments.

To evaluate the effect of rGDF11 (PEPROTECH, 0.1 mg/kg, i.p.) in old mice, rGDF11 dissolved in phosphate-buffered saline was given 5 days after ischemia and continued for 5 days (group 3, and 4). For the vehicle-treated group, human IgG (R&D systems, 100 ug/kg, i.p.) was injected as described above. The dose of rGDF11 was chosen from earlier studies [7, 12]. BrdU (Sigma, 75 mg/kg, i.p.) was administered for 5 days starting at day 5 post-ischemia at an intraperitoneal site different from GDF11 injections.

The animals were randomized to treatment groups by block randomization. Investigators remained blinded to the treatment and group allocations during the experiments and data analysis. Only animals that reached the experimental endpoint (30 days) were included in the study. Animals that died before initiation of treatments were excluded (n=6). The reason for death was a hemorrhagic transformation as reported earlier [53]. Three mice died on day 1 post-MCAo, one mouse died on day 2 and two mice died on day 3.

### Behavioral assessment

### Neurological deficit score (NDS)

The NDS is a 5-point stroke severity scale [52]. NDS is scored from 0 to 4 with four being the highest level of deficit and 0 being no deficit detected.

### Digigait testing

Digigait testing was performed and analyzed using the digigait ventral plane videography system (Mouse Specifics, Inc. Framingham, MA). The digigait ventral plane videography system consists of a transparent treadmill with a camera mounted beneath. Mice were placed on the treadmill briefly for 5-seconds before the treadmill was started at a speed of 1 cm/ second at an incline of zero degrees. The incline was kept consistent at zero degrees but the speed of the treadmill was increased by 1 cm/ second every 5-seconds until a final speed of 5 cm/ second was reached. The video camera mounted below recorded both the ability of the mouse to walk at the given speed and its gait. Once the test was completed, mice were placed in a recovery cage until all mice in their cage completed the task and then were returned to their home cage.

### Tail suspension test

The tail suspension was performed as described in [54]. After acclimatization to the testing room for one-hour, the mice were suspended 60 cm above a table by their tail and recorded for 6 minutes using a digital camera. After the recording was completed, the mice were carefully removed and placed into a recovery cage away from its cage-mates until all had undergone testing after which they were returned to their home cages. The videos were then analyzed by a trained observer who was blinded to surgical and treatment groups who recorded the total time the mouse spent immobile. The testing apparatus was cleaned with 70% ethanol between mice.

### Nest building score

The nest-building test was performed and analyzed as described earlier [31]. Mice were singly housed overnight in a cage on corncob bedding with one Nestlet, which is a cotton square, 2 inches X 2 inches (Lab Supply, Fort Worth, TX). Twelve hours later pictures were taken of the nests and scored using a 5-point scale. 1. The cotton square is untouched with over 90% still intact. 2. The cotton square is partially torn up but over 50% is still intact. 3. The cotton square is almost or completely torn up but no identifiable nest site is present (pieces scattered throughout the cage). 4. The cotton square is almost or completely torn up and there is an identifiable nest site but the sides of the nest are flat on more than 50% of its circumference. 5. The cotton square is almost or completely torn up and there is an identifiable nest site where the walls of the nest are higher than the body of the mouse on all sides.

### Open field-testing

Locomotor activity was assessed as described previously at day 30 after MCAo [55]. Mice were placed in a brightly lit box, 50cm wide x 50cm length x 38 height and allowed to explore freely for 10 minutes. The mice were filmed from above throughout the test. These videos were then analyzed using Noldus Ethovision behavior software (Leesburg, VA). Between mice, the boxes were cleaned with 70% ethanol.

### MRI testing

Mice were scanned on a Bruker 9.4T/Avance, 20 cm bore MRI system outfitted with microgradients and Paravision 5.1 software. Brain images were collected using a TURBORARE imaging sequence with a TR = 2500, a TE = 36 and NA = 2. The field of view was 3 cm and the slice thickness was 500 m and the matrix was set at 256 x 256. We utilized OsiriX^®^ MD.

### Western blot analysis

Mice were euthanized, transcardially perfused with 60mL cold sterile phosphate-buffered saline (PBS) and the brains were harvested. The olfactory bulb, brainstem, and cerebellum were dissected as described previously [56] and the cortex was removed for further process. The mouse brain sections were homogenized using lysis buffer (1%NP-40, 1mM phenylmethylsulfonyl fluoride, protease inhibitors; complete and miniphosStop tablets) and the supernatant was resolved on 4-20% gradient SDS –PAGE and transferred to polyvinylidene difluoride membrane. Protein concentration was confirmed by BCA Protein Assay Kit (Thermo Fisher Scientific Inc, Rockford IL). Blots were blocked with 5% bovine albumin serum for an hour and incubated with GDF11/8 (1:1000, Ab124721), and pSmad 2/3 (1:1000, D27F4) overnight at 4^0^C. Beta-actin was used as a loading control. Secondary antibodies (1:10,000) were diluted and the blots were incubated for 1 hour, and an ECL detection kit was used for signal detection.

### ELISA and multiplex

The brains from young and older mice were collected, and ELISA was performed following the manufacturer’s instructions (MyBioSource; MBS2021645) to determine GDF11 levels. This assay kit has high sensitivity and specificity for GDF11. To assess cytokines in the brain, a multiplex (Bio-Plex Pro^TM^ mouse cytokine 9-Plex Assay #MD000000EL) was performed following the manufacturer’s instructions.

### Assessment of brain tissue loss

After transcardial PBS and paraformaldehyde 4% perfusion, the brains were harvested from mice in group 3. The olfactory bulb, brainstem, and cerebellum were removed. The brain was post-fixed overnight in 4% PFA and placed in 30% sucrose solution for 48 hours before processing. The brains were then cut into 30-μm sections on a freezing microtome and every eighth slice was stained by cresyl violet to visualize tissue loss [22562356]. The slices were photographed, tissue atrophy was analyzed using computer software (Sigma scan Pro5) as previously described [57]. Tissue atrophy percentage was calculated by using the following formula: percentage tissue atrophy = (total ipsilateral tissue/total contralateral tissue) × 100.

### Assessment of CSF

After transcardial PBS and paraformaldehyde 4% perfusion, the brains were harvested from mice. The head was removed, skin, muscle, ears, nose tip and lower jaw were removed to expose the skull. The head was fixed in 4% PFA at 4^0^C. The head was then transferred to 40 ml of 0.01% sodium azide in PBS and rocked for 7 days at 4^0^C. The head was then transferred to a solution of 5mM gadopentetate dimeglumine (Bayer HealthCare Pharmaceuticals Inc., Wayne, NJ) and 0.01% sodium azide in PBS and rocked for 21 days at 4^0^C. We utilized OsiriX^®^ MD software to determine the areas of the ventricles and cerebrospinal fluid (CSF) of the mouse brains. Briefly, DICOM files of the acquired MRI data were imported into OsiriX. The whole brain was segmented as well as the regions of CSF and the ventricle areas ipsilateral and contralateral to the injury were identified and traced to obtain the CSF area.

### Corpus callosum area analysis

Three consecutive sections were taken from the MRI images (+0.48, +0.72+ 1.92 mm from bregma). The corpus callosum (CC) was identified and quantification was performed by an investigator blinded to treatment groups. The area from contralateral and ipsilateral mouse brain was quantified using ImageJ. The CC area was normalized to contralateral CC. The entire analysis was repeated twice to ensure reproducibility of the results.

### Immunohistochemistry (IHC) analysis

For estimation of gliosis, angiogenesis, and neurogenesis, mice in group 3 (n=5-6) were used. For the mouse brain, a standard procedure was utilized for IHC staining on 30-μm sections mounted on Fischer Scientific Superfrost Plus charged slides. Briefly, the tissue sections were rinsed in 0.1 M phosphate-buffered saline (PBS) pH 7.4. Antigen retrieval was performed by heating the tissue in a 10 mM sodium citrate buffer at pH 6.0. Tissue sections were incubated for 1 hour in blocking solution (0.1% Triton-X, 10% normal goat serum in 1X PBS) and then incubated overnight in primary antibody at 4°C (GDF11/8 1:250; IBA-1(Fujifilm Wako pure chemical corporation, NCNP24) 1:300; GFAP-Cy3 (Sigma-Aldrich C9205) 1:300; CD 31 (ab28364) 1: 100), DyLight 594 labelled Lycopersicon Esculentum (tomato) lectin (Vector Laboratories)1:100, BDNF (1:100), MBP (1:100, cell signaling technologies, 78896S), synaptophysin (1:300, ab14692).

### BrdU staining

Following three washes with 0.1M PBS, pH 7.4 for 10 minutes, sections were placed in ice-cold 1N hydrochloric acid (HCl) for 10 minutes, followed by incubation with 2N HCl for 30 minutes at 37^0^C. After 30 minutes, the sections were rinsed three times with 0.1M 1X PBS and then sections were incubated with 0.1M boric acid for 10 minutes. The sections were washed three times and blocked with blocking solution and incubated with primary antibodies (Doublecortin (Cell signaling Technology, 4604S) 1:200 and anti-BrdU (ab6326) 1:25) overnight at 4^0^C as described previously. Following overnight incubation with primary antibodies, tissue sections were washed with PBST (0.1% tween 20 in 1X PBS) and then incubated with the appropriate conjugated secondary antibody (1:500, Molecular Probes, Invitrogen) for 2 hours at room temperature except for GFAP treated tissue. After washing in PBST, sections were coverslipped with mounting media (VECTASHIELD Antifade Mounting Medium with DAPI, Vector Laboratories).

Three coronal brain sections per mouse, taken 0.02, 0.45, and 0.98 mm from bregma, were stained and visualized for quantification at 20X magnification at the core/penumbra junction to quantify positive cells. Additionally, the expression of synaptophysin was quantified as described earlier [58]. Furthermore, immunoreactivity for MBP and GFAP was analyzed in CC and peri-infarct striatum [59]. The average numbers of cells visualized from three adjacent regions at the core/penumbra junction were recorded for each mouse and quantification was done using Image J software. For the quantification of vessel percentage area, a total number of junctions and total vessel length Angio Tool was used. To assess the number of DCX and BrdU positive neurons, 5 hippocampal sections, every fifth section (from bregma-1.46 mm to bregma-2.30 mm) were used. Three images were taken from each mouse brain and values were totaled. Similarly, three sections of SVZ per mouse brain were imaged and the values were totaled.

### IHC for human tissue

All brain sections were from the mid-frontal cortex from the middle cerebral artery territory as determined by expert neuropathological assessments of H&E stained sections. The sections were from young controls [< 60 years=9, females (2) and males (7)], aged control [> 75 years, n=8, females (4) and males (4)]. Immunofluorescence staining was performed on the paraffin-embedded human brain tissue as described previously [60]. Immunohistochemically staining was performed by conventional deparaffinization of the human brains. Deparaffinized brain sections were incubated with 3% hydrogen peroxide for 15 minutes to reduce the background staining. Sections were washed three times with wash buffer (0.1% Triton-X in 1X PBS). The sections were blocked with blocking buffer (0.1% Triton-X, 10% fetal calf serum in 1X PBS) for an hour and incubated overnight with GDF11/8 primary antibody (1:200, Abcam) at 4^0^C. Samples were washed and subsequently incubated with Histofine (anti-rabbit secondary antibody fab fragment from Nichirei, Tokyo, Japan) for 30 min at RT. Immunoperoxidase staining was performed with the diaminobenzidine substrate kit (Nichirei, Tokyo, Japan) and counterstained with hematoxylin. For quantification, six images (20X and 40X) from the cortex of each human brain were taken on the Leica microscope using bright field and GDF11/8 immunoreactivity was quantified by scoring. Scoring was performed as follows: positively stained cells per section (40X) were scored as described, less than two cells = zero; three to five cells = 1; six to eight cells = 2; nine to twelve cells = 3; thirteen and above cells = 4. Data were analyzed by a blinded investigator.

### Statistical analysis

Data are presented as mean ±SEM except for NDS, which was presented as median (interquartile range) and analyzed with the Mann-Whitney test. Grubbs test was used to analyses outliers. Two-group comparisons were analyzed by the Student t-test. Mortality was analyzed by generating Kaplan-Meier survival curves and performing a Mantel-Cox log-rank test. Statistical significance was set at p<0.05.

## Supplementary Material

Supplementary Mathods

Supplementary Figures
