# Supplementary material for: Growth differentiation factor-11 supplementation improves survival and promotes recovery after ischemic stroke in aged mice
Source: Aging (Albany NY). 2020 May 4;12(9):8049–66. doi: 10.18632/aging.103122 (PMC7244081; doi:10.18632/aging.103122)
Supplement: Supplementary Mathods [file aging-12-103122-s001..pdf]

SUPPLEMENTARY METHODS

Middle cerebral artery occlusion (MCAo) and treatment

Briefly, transient focal ischemia was induced under isoflurane anesthesia by occlusion of the right middle cerebral artery (MCA) by a monofilament for 60 minutes. The monofilament was not inserted into the MCA in sham mice. The right common carotid artery (CCA) was identified and tied with a loose surgical knot. The external common artery (ECA) was identified and a knot was tied to block blood flow. The internal carotid artery (ICA) was tied with a loose knot with a silk suture. A small incision was made in the ECA and silicon-coated monofilament was introduced into the CCA. The monofilament was tied with a slip knot on the ECA, the ECA was gently retracted perpendicular to the ICA and the knot on the ICA was untied. By gentle maneuvering, the monofilament was introduced into the MCA and tied to avoid movement of the monofilament. After 60 minutes of occlusion, the monofilament was withdrawn from the ICA, and the ECA was cauterized and the knot on the CCA was untied to allow reperfusion. The suture diameter was 0.23 mm diameter for aged animals with a silicone coated tip at a length 5-6 mm.

Post MCAo care

All mice were administered 1 ml of 0.9% normal saline SC at two different injection sites daily for 7 days twice a day. The animals were provided with fresh wet mesh daily for the first week post-surgery. The wet mash was prepared by soaking the food pellets in distilled water until the consistency was soft and placed in shallow containers on the floor of the cage to ensure that the mice had access to food without having to rear upwards to obtain chow. For the first week, mice were only handled to measure body weights, administer saline, and to perform a brief neurological deficit score (NDS). The NDS was performed by gently placing the mouse in the new cage and neurological scores were recorded. This avoids unnecessary animal handling that could contribute to stress and mortality.
