# Supplementary material for: Growth differentiation factor-11 supplementation improves survival and promotes recovery after ischemic stroke in aged mice
Source: Aging (Albany NY). 2020 May 4;12(9):8049–66. doi: 10.18632/aging.103122 (PMC7244081; doi:10.18632/aging.103122)
Supplement: Supplementary Figures [file aging-12-103122-s002..pdf]

SUPPLEMENTARY FIGURES

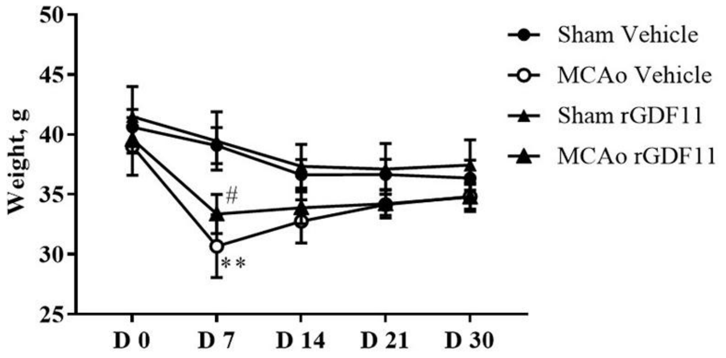

Supplementary Figure 1. Changes in body weight after the vehicle and GDF11 treatment. n=6-8, #p<0.05 Vs sham rGDF11, **p<0.01 Vs sham vehicle, treated group.

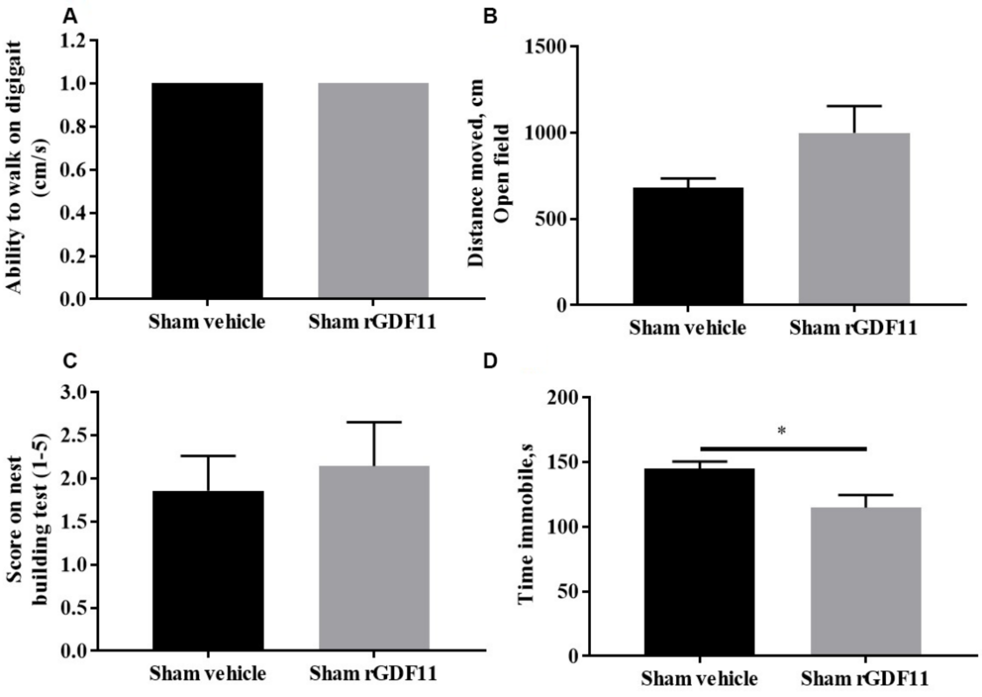

Supplementary Figure 2. No difference in (**A**) ability to walk on digigait, (**B**) distance moved in the open field and (**C**) nest-building activity was observed between sham vehicle and sham rGDF11 treated mice, and (**D**) decrease in time immobile was observed in the sham rGDF11 treated mice at day 30. *p<0.05, n=4-8.

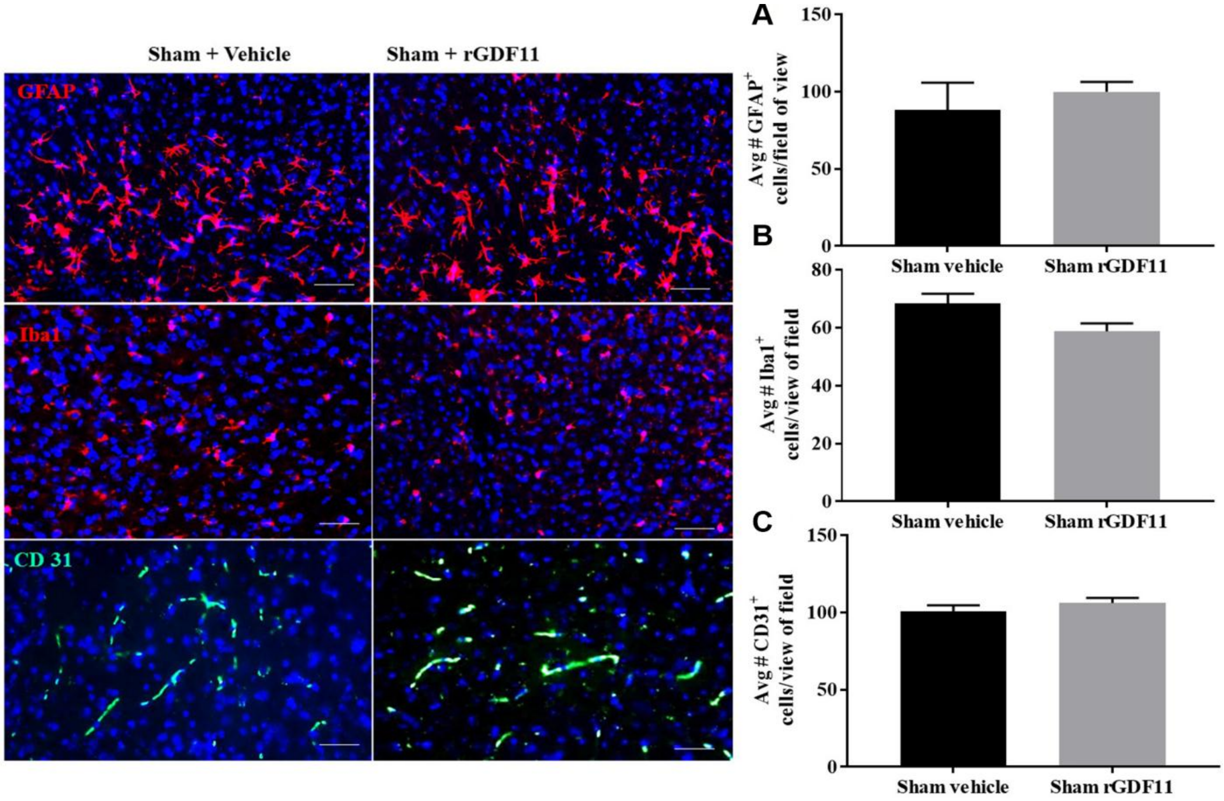

Supplementary Figure 3. Representative images and graphs showing average number (Avg#) of (**A**) GFAP, (**B**) Iba-1 and (**C**) CD31^{+} cells in sham vehicle and sham rGDF11. Magnification 20X. n=3-5.

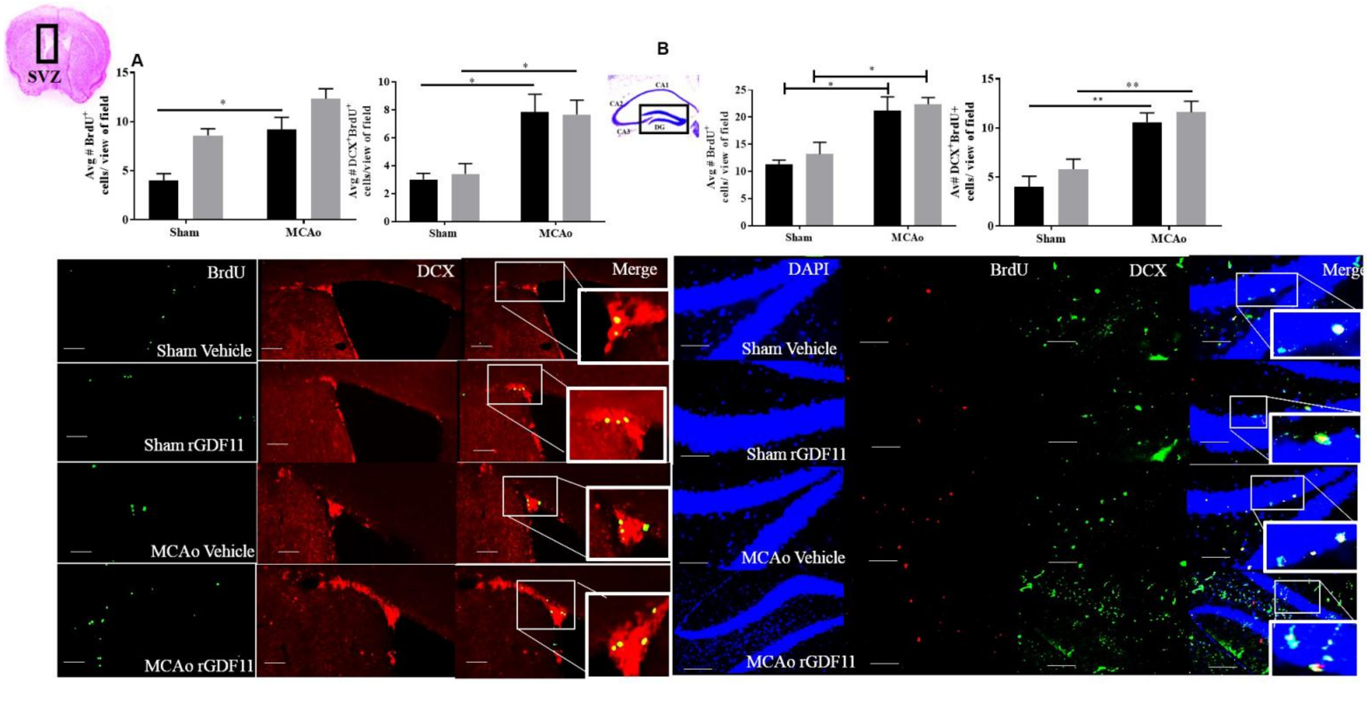

Supplementary Figure 4. Representative images and graphs showing BrdU and DCX staining in (**A**) SVZ and (**B**) hippocampus in sham and MCAo groups. Magnification 20X. n= 4-7, **p<0.01, *p<0.05.
